# Objectively measured physical activity, sedentary behaviour and all-cause mortality in older men: does volume of activity matter more than pattern of accumulation?

**DOI:** 10.1136/bjsports-2017-098733

**Published:** 2018-02-12

**Authors:** Barbara J Jefferis, Tessa J Parsons, Claudio Sartini, Sarah Ash, Lucy T Lennon, Olia Papacosta, Richard W Morris, S Goya Wannamethee, I-Min Lee, Peter H Whincup

**Affiliations:** 1 Department of Primary Care and Population Health, University College London, London, UK; 2 Population Health Sciences, Bristol Medical School, University of Bristol, Bristol, UK; 3 Harvard Medical School, Brigham and Women’s Hospital, Boston, Massachusetts, USA; 4 Population Health Research Institute, St George’s University of London, London, UK

**Keywords:** physical activity, sedentary behaviour, accelerometer, mortality, bouts.

## Abstract

**Objectives:**

To understand how device-measured sedentary behaviour and physical activity are related to all-cause mortality in older men, an age group with high levels of inactivity and sedentary behaviour.

**Methods:**

Prospective population-based cohort study of men recruited from 24 UK General Practices in 1978–1980. In 2010–2012, 3137 surviving men were invited to a follow-up, 1655 (aged 71–92 years) agreed. Nurses measured height and weight, men completed health and demographic questionnaires and wore an ActiGraph GT3x accelerometer. All-cause mortality was collected through National Health Service central registers up to 1 June 2016.

**Results:**

After median 5.0 years’ follow-up, 194 deaths occurred in 1181 men without pre-existing cardiovascular disease. For each additional 30 min in sedentary behaviour, or light physical activity (LIPA), or 10 min in moderate to vigorous physical activity (MVPA), HRs for mortality were 1.17 (95% CI 1.10 to 1.25), 0.83 (95% CI 0.77 to 0.90) and 0.90 (95% CI 0.84 to 0.96), respectively. Adjustments for confounders did not meaningfully change estimates. Only LIPA remained significant on mutual adjustment for all intensities. The HR for accumulating 150 min MVPA/week in sporadic minutes (achieved by 66% of men) was 0.59 (95% CI 0.43 to 0.81) and 0.58 (95% CI 0.33 to 1.00) for accumulating 150 min MVPA/week in bouts lasting ≥10 min (achieved by 16% of men). Sedentary breaks were not associated with mortality.

**Conclusions:**

In older men, all activities (of light intensity upwards) were beneficial and accumulation of activity in bouts ≥10 min did not appear important beyond total volume of activity. Findings can inform physical activity guidelines for older adults.

Nearly all epidemiological evidence used to estimate the shape of the dose–response curve between physical activity (PA) and mortality is based on self-reported PA.^1^ Moderately active compared with inactive adults have 20%–30% reductions in all-cause mortality, with greater reductions in older (>65 years) than middle-aged adults.^2^ PA is a key determinant of longevity globally.^3^ Current activity guidelines suggest accumulating ≥150 min moderate to vigorous PA (MVPA) per week in bouts lasting ≥10 min.^4 5^ The 10 min bout requirement was based on trial data for cardiometabolic risk factors only, not clinical end points.^5^ In order to test whether the accumulation of MVPA in ≥10 min bouts affects risk of mortality, prospective cohort studies with device-measured physical activity (which can provide minute by minute data for calculation of bouts) and mortality data are required, but few studies have such data. Such data can also inform whether accruing sedentary time in prolonged bouts is associated with adverse effects on mortality, as this has been identified as an important research gap.^6^ Many studies report that higher levels of self-reported sedentary time are associated with mortality,^7–10^ although self-reported sedentary behaviours may suffer from measurement error or recall bias.^11–15^ Experimental studies suggest benefits of breaking up sedentary time for metabolic and haemostatic markers.^16 17^ Hence, activity guidelines now suggest avoiding ‘long’ sedentary periods, but without quantifying how ‘long’ is detrimental.^4^

Recently, prospective cohort studies using body-worn devices to measure PA report that more time spent in MVPA is associated with lower mortality risks and sedentary behaviour with higher risks.^18–28^ However, few address the question of pattern of accumulation of activity rather than total volume. Most of the studies use the US National Health and Nutrition Examination Survey (NHANES) data set,^18–24^ and not all findings are consistent.^18 23^ There is little information from other populations and older age groups, >80 years.

We address important gaps in knowledge by focusing on older men: older adults are increasingly important given global population ageing. We use a community-dwelling cohort of older British men to investigate how device-measured PA is associated with all-cause mortality (including light PA (LIPA) and sedentary behaviour which are the predominant activities in this age group^29^). Importantly, we fill a research gap by investigating dose–response associations,^6^ testing for linear and non-linear associations in order to understand whether the reductions in mortality risk for higher levels of physical activity are linear, or if there is a threshold level at which the benefits per unit of activity decrease (and conversely for sedentary behaviour). We also investigate whether, as suggested elsewhere,^30^ the association of sedentary behaviour with mortality depends on PA level. Finally, a particularly novel and policy-relevant aspect of this paper is that we investigate patterns of accumulation of activity (including bout length and sedentary breaks) in relation to mortality. Answers to these questions will help inform future guidelines for older adults.

## Methods

### Sample

The British Regional Heart Study is a prospective cohort study of 7735 men recruited from a single general practice in each of 24 British towns in 1978–1980 (ages 40–59 years). In 2010–2012, survivors (n=3137) were invited to a physical examination.^31^

### Measurements at 2010–2012 examination

#### Objective physical activity assessment

Men wore a GT3x accelerometer (ActiGraph, Pensacola, FL, USA) over the right hip for 7 days, during waking hours, removing it for bathing and swimming (2% reported swimming). Data were processed using standard methods described previously.^29^ Non-wear time was excluded using the R package ‘Physical Activity’.^29 32^ By convention, we defined valid wear days as ≥600 min wear time, and included participants with ≥3 valid days. Each minute of activity was categorised using intensity threshold values of counts per minute (CPM) developed for older adults: <100 for sedentary behaviour (<1.5 Metabolic Equivalent of Task (MET)), 100–1040 for light activity (LIPA) (1.5–3 MET) and >1040 for MVPA (≥3 MET).^33^

#### Body mass index

Body mass index (BMI, kg/m^2^) was calculated from nurse-measured height (Harpenden stadiometer) and weight in light indoor clothing (Tanita body composition analyser (BC-418-MA)).

#### Questionnaire data

Men’s self-reported information included: current cigarette smoking, alcohol consumption, usual duration of night-time sleep, whether they lived alone and had pre-existing cardiovascular disease (CVD) (ever received a doctor diagnosis of heart attack, heart failure or stroke (with symptoms lasting >24 hours)). Mobility disability was present if the men reported being unable to do any of: (1) walking 200 yards without stopping and without discomfort; (2) climbing a flight of 12 stairs without holding on and taking a rest; or (3) bending down and picking up a shoe from the floor. Social class was based on longest held occupation at study entry (1978–1980) and categorised as manual and non-manual for parsimony (sensitivity analyses used the full seven categories of occupation and four categories of age leaving education). Region of residence (1978–1980) was grouped into Scotland, North, Midlands and South of England.

#### Mortality

Men were followed-up for all-cause mortality through National Health Service central registers until 1 June 2016.

### Patient involvement

Participants had the opportunity to contribute their views on future research priorities for the study, and detailed feedback about physical activity levels from the accelerometer study was given on request. A summary of the findings of the study and update on progress of the accelerometer study was mailed to the participants yearly.

### Statistical methods

Means, medians or proportions of covariates selected a priori were calculated according to quartiles of time spent in MVPA and sedentary behaviour. Cox proportional hazards models were used to estimate the HRs for mortality according to (1) total steps per day and total daily minutes in (2) MVPA, (3) LIPA and (4) sedentary behaviour, measured in 2010–2012. Each activity measure was analysed (1) in quartiles and (2) as a continuous variable. To aid interpretation, HRs were estimated for each increase in 1000 steps, 30 min of sedentary behaviour or LIPA and 10 min of MVPA. Model 1 was adjusted for measurement-related factors (average accelerometer wear time (min/day), season of wear (warm, May to September or cold, October to April), age, region of residence). Model 2 additionally adjusted for: social class, living alone, duration of sleep, smoking status, alcohol consumption and BMI. Model 3 further adjusted for presence of mobility disability. Model 4 also adjusted for other intensity of PA to investigate whether (1) MVPA and sedentary behaviour and (2) MVPA and LIPA were associated with mortality independent of each other. Model 5 adjusted simultaneously for MVPA, LIPA and sedentary behaviour as continuous variables (partition model). The linearity of associations between each measure of PA and sedentary behaviour and mortality was tested by comparing linear models with quadratic models using a likelihood ratio test in Stata, based on a priori expectations. Where non-linear associations were detected, the shape of the non-linear association was estimated using penalised splines in R. The penalised spline is a non-parametric estimation method which makes few assumptions about the underlying shape of the association. Predicted values from spline models were plotted. The Akaike information criterion (AIC) was compared between linear and spline models.

We estimated the HR for mortality among men who accumulated ≥150 min MVPA/week (1) in bouts lasting ≥1 min and (1) in bouts lasting ≥10 min. For MVPA and LIPA, we also compared minutes in bouts lasting 1–9 min with minutes in bouts of ≥10 min, testing the difference in coefficients using a post hoc test. For sedentary behaviour, we compared bouts lasting 1–15 min, 16–30, 31–60 and over 61 min. We estimated the HR for mortality for the number of sedentary breaks per hour (defined as the interruption of a sedentary bout lasting >1 min by ≥1 min of LIPA or MVPA). The number of sedentary breaks per hour was split into quartiles for analysis, models were adjusted for total sedentary time. Sensitivity analyses (reported in the online supplementary appendix 1) investigated (1) the skewed distribution of MVPA, (2) the percentage of the day spent in each activity, (3) excluding the first year of follow-up and (4) excluding men with disability and pre-existing CVD, (5) including men with pre-existing CVD (6) confounding by socioeconomic status. Analyses were conducted in Stata V.14.2^34^ and R V.3.4.0.^35^

## Results

Of 3137 surviving men, 1566 (50%) agreed to participate and returned an accelerometer with data. Of these, 1528 (49%) had ≥600 min/day wear time on ≥3 days. 254 men with pre-existing heart attack, heart failure or stroke were excluded, leaving 1274 men. Participants’ mean age was 78.4 (range 71–92) years (table 1). Mean accelerometer wear time was 855 min/day, of which 616 min was in sedentary behaviour and 199 min in LIPA. MVPA minutes had a right-skewed distribution, median 33 min (IQR 16–56) (table 1). There were dose–response associations across quartiles of MVPA, where men who were more active compared with less active were younger, less likely to smoke cigarettes and had lower alcohol consumption, BMI and prevalence of mobility disability, and spent less time in sedentary behaviour (table 1). Similarly, dose–response associations, in the opposite direction, were observed over quartiles of sedentary behaviour (data not presented). The distribution of bouts spent in each activity intensity is presented in online supplementary table 1.

10.1136/bjsports-2017-098733.supp1 Supplementary Appendix 1

**Table 1:** Characteristics of British men without pre-existing CVD or heart failure, by quartile of daily minutes spent in MVPA, measured in 2010–2012 (n=1274)

Mean (SD) or % (n)	Quartile of MVPA (min/day)				P (trend)	All men	n
	1	2	3	4			
	0.4 to <3.1	≥3.1 to <30.8	≥30.8 to <53.5	≥53.5			
n	291*	308*	340*	335*			1274
Age (years)	81.0 (5.0)	78.7 (4.7)	77.8 (4.0)	76.5 (3.5)	<0.0001	78.4 (4.6)	1274
Manual social class, % (n)	52 (150)	45 (139)	45 (154)	46 (151)	0.29†	46.9 (594)	1266
Lives alone, % (n)	23 (65)	19 (59)	19 (62)	16 (52)	0.18†	19.0 (238)	1256
Smoker, % (n)	6.6 (19)	4.6 (14)	1.5 (5)	2.1 (7)	0.002‡	3.6 (45)	1257
Alcohol (units per week)	5.2 (7.3)	6.0 (7.7)	6.8 (7.5)	7.2 (7.9)	<0.0001	6.4 (7.6)	1240
BMI (kg/m^2^)	28.2 (4.6)	27.4 (3.6)	26.9 (3.6)	26.1 (3.1)	<0.0001	27.1 (3.8)	1263
Sleep per night (hours)	6.8 (1.5)	6.9 (1.4)	6.8 (1.3)	6.9 (1.2)	0.32	6.9 (1.4)	1245
Mobility disability present, % (n)	48.8 (139)	14.3 (44)	7.2 (24)	6.4 (21)	<0.0001	18.2 (228)	1253
Total activity (counts per minute)	61 669 (24 590)	113 645 (23 416)	171 554 (29 976)	294 370 (83 994)	<0.0001	164 749 (99 271)	1274
Steps/day	1895 (883)	3646 (832)	5302 (1022)	8401 (2370)	<0.0001	4938 (2794)	1274
% time spent sedentary	81.8 (6.7)	75.1 (5.6)	70.4 (5.7)	63.0 (7.5)	<0.0001	72.2 (9.3)	1274
% time LIPA	17.3 (6.5)	22.2 (5.5)	24.8 (5.7)	27.3 (6.5)	<0.0001	23.1 (7.0)	1274
% time MVPA	0.8 (0.4)	2.6 (0.6)	4.8 (0.8)	9.7 (3.1)	<0.0001	4.7 (3.7)	1274
Sedentary behaviour (min/day)	676 (76)	638 (65)	607 (68)	552 (76)	<0.0001	616 (84)	1274
LIPA (min/day)	144 (56)	189 (50)	214 (52)	239 (61)	<0.0001	199 (65)	1274
MVPA (min/day)	6.9 (3.7)	22.3 (4.8)	41.4 (6.5)	84.7 (26.9)	<0.0001	40 (33)	1274
Sedentary breaks (median, IQR)§	5.8 (4.6–6.8)	6.8 (5.9–7.9)	7.4 (6.5–8.7)	8.4 (6.9–9.6)	<0.0001	7.0 (5.9–8.5)	1274

*Maximum n in quartile varies slightly with missing covariate data.

†Pearson χ^2^ test.

‡Fisher’s exact test.

§Median and IQR of the number of breaks in sedentary time per hour.

BMI, body mass index; CVD, cardiovascular disease; LIPA, light physical activity; MVPA, moderate to vigorous physical activity.

### PA, sedentary behaviour and all-cause mortality

During a median follow-up of 5.0 years (range 0.2–6.1), 194 deaths occurred. For each additional 30 min in sedentary behaviour and LIPA or 10 min in MVPA, HRs for all-cause mortality (model 1) were respectively 1.17 (95% CI 1.10 to 1.25) (table 2), 0.83 (95% CI 0.77 to 0.90) (table 3) and 0.90 (95% CI 0.84 to 0.96) (table 4). For each additional 1000 steps/day the HR was 0.84 (95% CI 0.78 to 0.91) (table 5). Adjustments for sociodemographic factors, health behaviours and sleep time (model 2) and mobility disability (model 3) minimally affected the estimates and CIs. Adjustment for MVPA (model 4) did not meaningfully change associations for sedentary behaviour (table 2) or LIPA (table 3), but adjustment for sedentary time reduced the association for MVPA to 1.00 (95% CI 0.92 to 1.09) (table 4). In the partition model (model 5, tables 2–4), only LIPA was significant at HR 0.86 (95% CI 0.78 to 0.94 per 30 min/day) on mutual adjustment for MVPA, sedentary behaviour and sleep time. There were dose–response associations across quartiles of activity, with higher risk in higher quartiles of sedentary behaviour (table 2) and lower risk in higher quartiles of MVPA (table 4) and steps (table 5).

**Table 2:** Association between minutes per day in sedentary behaviour with all-cause mortality among 1181 British men without pre-existing CHD, stroke or heart failure

	Quartile 1 (295–560)	Quartile 2 (561–616)		Quartile 3 (617–672)		Quartile 4 (673–1054)		Total*	
Number of participants (n deaths)	296 (32)	302 (35)		294 (52)		289 (75)		1181 (194)	
Person-years	1461	1475		1413		1297		5646	
Mortality/1000 person-years	21.9	23.7		36.8		57.8		34.4	
		**HR**	**95% CI**	**HR**	**95% CI**	**HR**	**95% CI**	**HR**	**95% CI**
Model 1†	Reference	1.14	0.70 to 1.84	1.65	1.04 to 2.63	3.08	1.93 to 4.92	1.17	1.10 to 1.25
Model 2‡	Reference	1.19	0.73 to 1.93	1.71	1.06 to 2.73	3.18	1.96 to 5.15	1.17	1.10 to 1.26
Model 3§	Reference	1.16	0.71 to 1.88	1.58	0.98 to 2.54	2.80	1.71 to 4.57	1.15	1.07 to 1.23
Model 4¶	Reference	1.14	0.69 to 1.91	1.55	0.91 to 2.64	2.73	1.50 to 4.95	1.15	1.06 to 1.26
Model 5**	Reference							0.99	0.92 to 1.06

*HR for mortality per 30 min of sedentary behaviour per day (continuous variable).

†Model 1=age+region of residence+season of wear+accelerometer wear time.

‡Model 2=model 1+social class+alcohol use+smoking+sleep time+living alone+body mass index.

§Model 3=model 2+mobility disability.

¶Model 4=model 3+MVPA.

**Model 5=model 3+LIPA+ MVPA (but without adjustment for accelerometer wear time).

CHD, coronary heart disease; LIPA, light physical activity; MVPA, moderate to vigorous physical activity.

**Table 3:** Association between minutes per day in light physical activity with all-cause mortality among 1181 British men without pre-existing CHD, stroke or heart failure

	Quartile 1 (5–154)	Quartile 2 (155–197)		Quartile 3 (198–238)		Quartile 4 (239–472)		Total*	
Number of participants (n deaths)	284 (81)	298 (55)		300 (28)		299 (30)		1181 (194)	
Person-years	1249	1413		1495		1488		5646	
Mortality/1000 person-years	64.9	38.9		18.7		20.1		34.4	
		HR*	**95% CI**	**HR**	**95% CI**	**HR**	**95% CI**	**HR**	**95% CI**
Model 1†	Reference	0.66	0.46 to 0.94	0.35	0.23 to 0.55	0.47	0.29 to 0.94	0.83	0.77 to 0.90
Model 2‡	Reference	0.68	0.48 to 0.98	0.37	0.23 to 0.58	0.46	0.29 to 0.75	0.83	0.77 to 0.90
Model 3§	Reference	0.74	0.51 to 1.06	0.39	0.25 to 0.62	0.51	0.31 to 0.82	0.85	0.78 to 0.92
Model 4¶	Reference	0.76	0.53 to 1.10	0.42	0.27 to 0.68	0.57	0.34 to 0.95	0.87	0.80 to 0.95
Model 5**	Reference							0.86	0.78 to 0.94

*HR for mortality per 30 min of LIPA per day (continuous variable).

†Model 1=age+region of residence+season of wear+accelerometer wear time.

‡Model 2=model 1+social class+alcohol use+smoking+ sleep time+living alone+body mass index.

§Model 3=model 2+mobility disability.

¶Model 4=model 3+MVPA.

**Model 5=model 3+sedentary behaviour+MVPA (but without adjustment for accelerometer wear time).

CHD, coronary heart disease; LIPA, light physical activity; MVPA, moderate to vigorous physical activity.

**Table 4:** Association between moderate to vigorous physical activity with all-cause mortality among 1181 British men without pre-existing CHD, stroke or heart failure

	Quartile 1 (0.4–15)	Quartile 2 (16–32)		Quartile 3 (33–55)		Quartile 4 (56–187)		Total*	
Number of participants (n deaths)	297 (86)	296 (53)		292 (32)		296 (23)		1181 (194)	
Person-years	1321	1422		1432		1471		5646	
Mortality/1000 person-years	65.1	37.2		22.3		15.6		34.4	
		**HR**	**95% CI**	**HR**	**95% CI**	**HR**	**95% CI**	**HR**	**95% CI**
Model 1†	Reference	0.75	0.53 to 1.06	0.54	0.35 to 0.82	0.45	0.27 to 0.73	0.90	0.84 to 0.96
Model 2‡	Reference	0.76	0.53 to 1.08	0.54	0.35 to 0.84	0.45	0.27 to 0.75	0.90	0.84 to 0.96
Model 3§	Reference	0.88	0.60 to 1.28	0.63	0.40 to 0.99	0.52	0.31 to 0.87	0.92	0.86 to 0.98
Model 4¶	Reference	1.05	0.71 to 1.57	0.89	0.53 to 1.47	0.90	0.48 to 1.70	1.00	0.92 to 1.09
Model 5**	Reference							0.95	0.89 to 1.02

*HR for mortality per 10 min of MVPA per day (continuous variable).

†Model 1=age+ region of residence+season of wear+accelerometer wear time.

‡Model 2=model 1+social class+alcohol use+smoking+sleep time+living alone+body mass index.

§Model 3=model 2+mobility disability.

¶Model 4=model 3+sedentary behaviour.

**Model 5=model 3+sedentary behaviour+LIPA (but without adjustment for accelerometer wear time).

CHD, coronary heart disease; LIPA, light physical activity; MVPA, moderate to vigorous physical activity.

**Table 5:** Association between steps per day with all-cause mortality among 1181 British men without pre-existing CHD, stroke or heart failure

	Quartile 1 (121–2927)	Quartile 2 (2928–4532)		Quartile 3 (4533–6412)		Quartile 4 (6413–17 781)		Total*	
Number of participants (n deaths)	293 (93)	295 (45)		299 (39)		294 (17)		1181 (194)	
Person-years	1281	1428		1467		1470		5646	
Mortality/1000 person-years	72.6	31.5		26.6		11.6		34.4	
		**HR**	**95% CI**	**HR**	**95% CI**	**HR**	**95% CI**	**HR**	**95% CI**
Model 1†	Reference	0.56	0.39 to 0.81	0.53	0.36 to 0.79	0.29	0.17 to 0.51	0.84	0.78 to 0.91
Model 2‡	Reference	0.63	0.43 to 0.93	0.59	0.39 to 0.90	0.31	0.17 to 0.57	0.84	0.78 to 0.91
Model 3§	Reference	0.63	0.43 to 0.93	0.59	0.39 to 0.90	0.31	0.17 to 0.57	0.86	0.80 to 0.93

*HR for mortality per 1000 steps per day (continuous variable).

†Model 1=age+ region of residence+season of wear+accelerometer wear time.

‡Model 2=model 1+social class+alcohol use+smoking+sleep time+living alone+body mass index.

§Model 3=model 2+mobility disability.

CHD, coronary heart disease.

### Shape of associations

Likelihood ratio tests suggested better fit for quadratic than linear models of step count or MVPA minutes (both P<0.001) and all-cause mortality. In models for steps and MVPA, the increment in goodness of fit (based on AIC) between linear and spline models was minimal (online supplementary table 2). Plots of estimated splines (online supplementary figures 1 and 2) did not show great deviations from linearity. Hence, for clinical interpretation, the simpler linear model was adequate.

### Bouts of activity and all-cause mortality

Table 6 presents the HR for mortality for each minute of MVPA spent in bouts; the HR per minute of MVPA spent in bouts lasting 1–9 min was 0.99 (95% CI 0.98 to 1.00), and 0.99 (95% CI 0.98 to 1.01) per minute of MVPA spent in bouts lasting ≥10 min; HRs did not differ (post hoc test P=0.59). Equivalent estimates for LIPA were HR 0.99 (0.99, 1.00) and 1.00 (0.99, 1.01), respectively (HRs did not differ; post hoc test P=0.48). Adjusting for presence of mobility disability attenuated HRs.

**Table 6:** Association between duration of bouts of sedentary behaviour, LIPA and MVPA* with all-cause mortality among 1181 British men without pre-existing CHD, stroke or heart failure

	Bouts of 1–9 min		Bouts of ≥10 min						P (no difference)†
MVPA	HR ‡	95% CI	HR ‡	95% CI	HR ‡	95% CI	HR ‡	95% CI	
Model 1§	**0.99**	**(0.98 to 1.00)**	0.99	(0.98 to 1.01)					0.594
Model 2¶	**0.99**	**(0.98 to 1.00)**	0.99	(0.98 to 1.01)					
Model 3**	0.99	(0.98 to 1.00)	0.99	(0.98 to 1.01)					
LIPA									
Model 1§	**1.00**	**(1.00 to 1.00)**	1.00	(0.99 to 1.01)					0.482
Model 2¶	**1.00**	**(1.00 to 1.00)**	1.00	(0.99 to 1.01)					
Model 3**	**1.00**	**(1.00 to 1.00)**	1.00	(0.99 to 1.01)					
Sedentary behaviour	1–15 min		16–30 min		31–60 min		>61 min		
Model 1§	**1.01**	**(1.00 to 1.01)**	**1.01**	**(1.00 to 1.01)**	**1.01**	**(1.00 to 1.01)**	**1.01**	**(1.00 to 1.01)**	0.290
Model 2¶	**1.01**	**(1.00 to 1.01)**	**1.01**	**(1.00 to 1.01)**	**1.01**	**(1.00 to 1.01)**	**1.01**	**(1.00 to 1.01)**	
Model 3**	**1.01**	**(1.00 to 1.01)**	**1.01**	**(1.00 to 1.01)**	**1.01**	**(1.00 to 1.01)**	**1.01**	**(1.00 to 1.01)**	

Bold values denote P<0.05.

*The number of min/day in bouts of the specified duration. HR is per minute of activity.

†Post hoc test for no difference between bout durations.

‡HR per minute in bout of specified duration.

§Model 1=age+region of residence+season of wear+accelerometer wear time+minutes of sedentary behaviour.

¶ Model 2=model 1+social class+alcohol use+smoking+sleep time+living alone+body mass index.

** Model 3=model 2+mobility disability.

CHD, coronary heart disease; LIPA, light physical activity; MVPA, moderate to vigorous physical activity.

The HR for accumulating 150 min MVPA/week in sporadic minutes (achieved by 66% of men) was 0.59 (95% CI 0.43 to 0.81) in model 1, and was not meaningfully changed in models 2 and 3 (data not presented). The HR for accumulating 150 min MVPA/week in bouts lasting ≥10 min (achieved by 16% of men) was 0.58 (95% CI 0.33 to 1.00) in model 1, and changed little in model 3. The model for ‘meeting the guidelines in bouts of ≥1 minute’ (yes/no) is not adjusted for total MVPA time per week, because the binary variable cuts the total MVPA time per week at 150 min/week, so the two are highly correlated (r>0.8).

The numbers of minutes spent in sedentary bouts lasting 1–15 min, 16–30, 31–60 and >61 min were all similarly associated with mortality; each HR 1.01 (95% CI 1.00 to 1.01) per minute fully adjusted (table 6). Analyses of number of sedentary breaks found that the HR for mortality among men in higher quartiles did not differ compared with the lowest quartile (table 7). See online supplementary appendix 1 for results of sensitivity analyses.

**Table 7:** Association between number of sedentary breaks per hour* with all-cause mortality among 1181 British men without pre-existing CHD, stroke or heart failure

	Quartile 1 (0.3–5.7)	Quartile 2 (5.8–6.9)		Quartile 3 (7.0–8.4)		Quartile 4 (8.5–15.9)		Total
Number of participants (n deaths)	275 (64)	305 (64)		297 (38)		304 (28)		1181 (194)
Person-years	1243	1428		1472		1504		5646
Mortality/1000 person-years	51.5	44.8		25.8		18.6		34.4
		**HR†**	**95% CI**	**HR†**	**95% CI**	**HR†**	**95% CI**	
Model 1‡	Reference	1.28	0.86 to 1.92	1.04	0.61 to 1.76	1.22	0.61 to 2.42	
Model 2§	Reference	1.21	0.81 to 1.81	0.95	0.56 to 1.62	1.06	0.53 to 2.11	
Model 3¶	Reference	1.22	0.81 to 1.82	0.95	0.56 to 1.61	1.01	0.50 to 2.02	

*A sedentary break is the interruption of a sedentary bout lasting >1 min by ≥1 min of LIPA or MVPA.

†HR is per quartile of sedentary breaks per hour.

‡Model 1=age+ region of residence+season of wear+accelerometer wear time+minutes of sedentary behaviour.

§Model 2=model 1+social class+alcohol use+smoking+sleep time+living alone+body mass index.

¶Model 3=model 2+mobility disability.

CHD, coronary heart disease; LIPA, light physical activity; MVPA, moderate to vigorous physical activity.

## Discussion

Among community-dwelling older men, we observed consistent prospective associations between higher total daily step count, minutes spent in LIPA or MVPA, lower sedentary time and lower risk of all-cause mortality. Associations changed little after adjustment for other health behaviours, BMI, presence of mobility disability and wear time. Associations of LIPA with mortality were only slightly further attenuated after adjustment for time spent in sedentary behaviour and MVPA, although associations between MVPA and mortality were entirely attenuated after adjustment for sedentary behaviour. The lower mortality risks were gained across the spectrum of activity levels, not confined to a particular threshold level. The total volume rather than pattern of accrual of physical activity was the most important influence on mortality.

Our data extend evidence to an older population (range 72–91 years at baseline), which is important as data on the over 80s are sparse,^25^ and to a non-US population (most reports use US data,^18–25 27 28^ nearly all use one data source). Few studies of device-measured activity and mortality have looked at light activity,^21 36^ or tested non-linearity in activity–mortality associations,^24 26 27^ and only one investigated bouts of MVPA,^23^ whereas we look at specific bouts of MVPA, LIPA, sedentary behaviour as well as the number of breaks in sedentary time.

### PA intensity and duration

Overall in our older aged sample of men, the associations between PA and mortality tended to be stronger than in younger adults, in line with findings of a meta-analysis of self-reported PA in relation to mortality.^2^ Comparing our findings with other studies with objective PA data is difficult because definitions of activity intensity and analysis methods vary. We found that each 30 min/day increase in sedentary behaviour was associated with a 15% increase in mortality risk, after exclusion of men with pre-existing CVD and exclusion of the first year of follow-up data. However, the adjustments for LIPA and MVPA in the partition model fully attenuated the association. While an early NHANES study reported that accelerometer-measured sedentary behaviour was associated with incident mortality,^18^ a study with longer follow-up and excluding prevalent CVD and deaths in the first 2 years of follow-up did not find significant associations.^23^ Additionally, a recent study of older women found that the raised risks of mortality associated with higher sedentary time were fully attenuated after adjusting for MVPA.^28^

In our study, each 30 min/day increase in LIPA was associated with a 17% reduction in mortality, which was robust to adjustment for sedentary behaviour and MVPA, suggesting that the increase in LIPA rather than the reduction in sedentary behaviour was most important. In a younger NHANES sample, a reduction in mortality of 16% was found per hour of LIPA.^36^ They defined LIPA as >2020 CPM (compared with >1040 CPM in our study), and did not adjust for MVPA or account for pre-existing disease.^36^ Another analysis of NHANES found a 17% reduction in mortality per hour of LIPA adjusted for MVPA, but using lower cut points (100–760 CPM).^24^ In contrast, a study of older women did not find that LIPA was associated with consistent reductions in mortality, although different definition of LIPA was used.^28^

We found that each 10 min/day increase in MVPA was associated with a 10% reduction in mortality (approximately 75% reduction per hour), which was not explained by adjustment for behavioural and social confounders and mobility disability whereas in NHANES data, the adjusted estimate was approximately 40% reduction per hour MVPA, but using a lower cut point (>760 CPM) to define MVPA.^24^ However, in models adjusting simultaneously for all intensities of activity, significant associations were observed only for LIPA, suggesting that among older men the lighter intensity stimulus is sufficient for prevention of mortality. The associations between LIPA and mortality were robust to adjustment for behavioural and social confounders and mobility disability, but future work should investigate the dose of activity that is protective against geriatric syndromes (such as cognitive and functional limitations), which may be on the pathway to raised risks of mortality and are increasingly important for elderly health and well-being.

We found that each increase of 1000 steps/day was associated with a 15% reduction in mortality, compared with a 6% reduction in the younger Australian and Tasmanian cohorts (average age <60 years at baseline).^26^

### Non-linearity of associations

Given the very marginal benefits of the non-linear models, we concluded that more steps, LIPA and MVPA and less sedentary behaviour are beneficial, rather than there being a particular threshold for benefits to accrue.

### Pattern of activity: bouts and breaks

Many PA guidelines advise accumulating MVPA in bouts lasting over 10 min and avoiding long spells of sedentary behaviour.^4 5^ If pattern of activity beyond total volume was important, we would expect the minutes spent in bouts of MVPA lasting ≥10 min to be more strongly associated with mortality than minutes spent in shorter bouts (and the same for shorter bouts of sedentary behaviour), but our findings did not support this. Furthermore, the benefit from accumulating 150 min sporadically was very similar to accumulating it in bouts ≥10 min. This suggests that total volume of PA rather than the pattern is important. Hence, for older men, for all-cause mortality at least, accumulating bouts of activity lasting ≥10 min may not be important. However, these analyses should be replicated in larger samples. We found no evidence that sedentary breaks were associated with mortality risk, once total sedentary time was accounted for, indicating that in this age group, total time rather than pattern of accumulation may be key. Taken together with the partition analysis results, this suggests that the guidelines for older adults may do better to focus on increasing time spent in light (or more intense) activity in order to gain the benefits of activity and by implication displace sedentary time, rather than encouraging breaks in sedentary periods as a means to reduce sedentary bout length. To our knowledge, only two other studies have directly examined bouts; one found that more time spent in MVPA bouts of ≥10 min was associated with lower mortality, but that sedentary bouts were not associated with mortality,^23^ and the other study concluded that longer sedentary bouts were associated with raised mortality risk.^27^

### Interactions of physical activity with other variables

It is suggested that the raised mortality risks associated with higher sedentary time are heightened in people with low MVPA levels.^30^ We did not find strong evidence to suggest this, but a stratified analysis suggested stronger associations between sedentary behaviour and mortality in the less active men. This is consistent with data from a meta-analysis including over 1 million individuals using self-reported PA, and sedentary behaviour found that the risks of sedentary behaviour were more pronounced in the less active individuals.^30^ Two analyses of device-measured activity in NHANES data reported similar patterns,^19 24^ but in a study of older adults, the reverse was found.^25^

### Strengths and limitations

This study benefits from prospectively collected data on exposures, important confounders and mediators and mortality. PA was measured using accelerometers and the PA intensities defined using age-appropriate and validated cut points.^33^ The sedentary behaviour measure does not include postural data and could include some standing time; however, hip-worn ActiGraph-measured sedentary behaviour has minimal bias compared with thigh-worn activPAL-measured sedentary behaviour (correlation r=0.76) in a sample of middle-aged adults.^37^ In a sample of healthy older adults, the ActiGraph cut point of <100 CPM has an estimated 93% sensitivity and 58% specificity; 11.8% of time classified by activPAL as standing was classified by accelerometer as sedentary^38^; however, in comparison, our sample is older and less healthy, so likely to engage in less prolonged standing time, which would improve classification. The response rate to the accelerometer study was similar or superior to other studies of older adults; nevertheless, participants were more often younger, and had healthier behaviours than non-participants, and may therefore have been more physically active and less sedentary than the general population. Data are from a population-based cohort of community-dwelling older predominantly white British men, so results may not apply to women, other ethnicities or younger men; however, other studies have not found evidence that the associations between PA or sedentary behaviour and mortality differ by gender^18 24 26 27 36^ and a recent study of older women finds associations in the same direction as ours, although due to methodological differences it is hard to compare effect sizes.^28^ Given that LIPA and sedentary time are highly correlated, it can be difficult to distinguish their effects; we did not use isotemporal substitution analyses as the sedentary behaviour and LIPA were too highly correlated, resulting in problems with collinearity and model convergence. In sensitivity analyses, our findings did not meaningfully change after exclusion of men with mobility disability, prevalent CVD and the first year of follow-up, suggesting findings are not likely to be due to reverse causality.

## Conclusions

The dose–response associations between sedentary behaviour and mortality as well as inverse associations between MVPA and LIPA suggest that among older men there are sustained benefits to longevity from physical activity of all intensities, from LIPA upwards. Results suggest that all activities, however modest, are beneficial. The finding that LIPA is associated with lower risk of mortality is especially important among older men, as most of their daily PA is light intensity. Furthermore, the pattern of accumulation of physical activity did not appear to alter the associations with mortality, suggesting that it would be beneficial to encourage older men to be active irrespective of bouts. Future work should replicate the investigation into bouts of activity in larger samples and including women. Given the rapid decline in physical activity with age among the oldest old populations, encouraging even light activity may provide benefits for longevity.

**What are the findings?**

- In older British men, accumulating more minutes of activity from light intensity upwards was associated with lower all-cause mortality.
- There was no evidence to suggest that accumulating moderate to vigorous activity in bouts lasting ≥10 min lowered risk of mortality compared with accumulating activity in shorter bouts, nor that breaking up sedentary time was associated with lower mortality risks.

**How might it impact on clinical practice in the future?**

Findings could refine physical activity guidelines and make them more achievable for older adults with low activity levels: stressing the benefits of all activities, however modest, from light intensity upwards; second, encouraging accumulating activity of all intensities without the need to sustain bouts of 10 min or more.
