# Supplementary material for: Objectively measured physical activity, sedentary behaviour and all-cause mortality in older men: does volume of activity matter more than pattern of accumulation?
Source: Br J Sports Med. 2018 Feb 12;53(16):1013–20. doi: 10.1136/bjsports-2017-098733 (PMC6691867; doi:10.1136/bjsports-2017-098733)
Supplement: Supplementary Appendix 1 [file bjsports-2017-098733supp001.docx]

**Web Appendix**

**Sensitivity analyses: Methods**

Analyses were repeated (i) using square root transformed MVPA as MVPA minutes were right skewed (ii) using percentage of the day in MVPA, LIPA and sedentary behaviour rather than minutes per day adjusted for wear time (iii) excluding the first year of follow up (during which there were 17 deaths), to reduce the risk of reverse causality and (iv) excluding men with mobility limitations, the first year of follow up and prevalent CVD (to reduce the risk of reverse causality) (v) including men with pre-existing CVD because CVD is common in older adults (17% of this sample), and adjusting for presence of CVD (vi) confounding by social class, replacing the manual vs non manual occupation variable with first the full 7 category occupation variable and second, with a 4 category variable age leaving education (8-15, 16-17, 18-21 and 22+ years). We investigated interactions firstly between sedentary behaviour and PA level, and secondly between each of MVPA, LIPA and sedentary behaviour and (i) age (above or below mean age of 78 years) and (ii) adiposity, above or below BMI of 28 Kg/m^2^.

**Sensitivity analyses: Results**

Analyses in Table 2 were repeated using square root transformed MVPA and the patterns of results observed were similar to untransformed data. Analyses using percentage of the day spent in MVPA, LIPA and sedentary behaviour rather than minutes per day adjusted for wear time, did not change results (data not presented).

In analyses excluding 17 men who died in the first year of follow-up, point estimates were similar to those in Table 2 and overall the pattern of results were the same. In analyses excluding 247 men with mobility disability and 254 with pre-existing CVD, point estimates were similar but a little weaker than in Table 2 for continuous measures of PA and sedentary behaviour, and for the analyses of quartiles, some confidence intervals included 1, but the overall pattern of results remained similar. A sensitivity analysis including 254 men with pre-existing CVD and adjusting for their conditions also found a similar pattern of results, however HRs were somewhat stronger. Analyses with 7 category occupation variable or years of education did not meaningfully or statistically differ from analyses using the 2 category occupational class variable.

We tested whether the associations between sedentary behaviour and mortality differed in the above median active (32 minutes MVPA per day) vs below median active and observed evidence of borderline statistical significance for an interaction (LRT, p=0.08); in stratified analyses the HR for mortality for 30 minutes of sedentary time was 1.23 (95%CI 1.13, 1.33) among men doing below median MVPA and HR 1.02 (95%CI 0.90, 1.16) in men doing above median MVPA. There was some evidence that the association between MVPA and mortality was stronger in the men aged above the mean age of 78 years than below (LRT, p=0.05), however there was no evidence for a difference using square root transformed MVPA. We did not find evidence that associations between mortality and LIPA or sedentary behaviour varied by age, or that associations with steps, MVPA, LIPA or sedentary behaviour varied by overweight status.

**Sensitivity analyses: Discussion**

We investigated interactions with (i) age and (ii) adiposity, and found some evidence that associations between MVPA and mortality were stronger in the men aged in their eighties than in their lower seventies, similar to the findings of Fishman who reported stronger associations in the over 80s, however not all studies find interactions with age^1-4^. In line with NHANES^5^ and Tasped^1^ studies, we did not find evidence that the association between sedentary behaviour and mortality varied by BMI, although other findings from NHANES reported that associations were stronger in adults with higher waist circumference^6^. Our sensitivity analyses with exclusion of the first year of follow up, pre-existing CVD and mobility disability and also inclusion of the men with CVD suggested that the pattern of our findings was robust. Likewise the lack of change of results when using more detailed occupational class and education variables, suggested that for these analyses, we had adequately captured socioeconomic status in our simple variable.

1. Dwyer T, Pezic A, Sun C, et al. Objectively Measured Daily Steps and Subsequent Long Term All-Cause Mortality: The Tasped Prospective Cohort Study. *PLoS One* 2015;10(11):e0141274. doi: 10.1371/journal.pone.0141274

2. Ensrud KE, Blackwell TL, Cauley JA, et al. Objective Measures of Activity Level and Mortality in Older Men. *J Am Geriatr Soc* 2014 doi: 10.1111/jgs.13101 [doi]

3. Fishman EI, Steeves JA, Zipunnikov V, et al. Association between Objectively Measured Physical Activity and Mortality in NHANES. *Med Sci Sports Exerc* 2016;48(7):1303-11. doi: 10.1249/MSS.0000000000000885

4. Loprinzi PD. Light-Intensity Physical Activity and All-Cause Mortality. *Am J Health Promot* 2016 doi: 10.4278/ajhp.150515-ARB-882

5. Matthews CE, Keadle SK, Troiano RP, et al. Accelerometer-measured dose-response for physical activity, sedentary time, and mortality in US adults. *Am J Clin Nutr* 2016;104(5):1424-32. doi: 10.3945/ajcn.116.135129

6. Schmid D, Ricci C, Baumeister SE, et al. Replacing Sedentary Time with Physical Activity in Relation to Mortality. *Med Sci Sports Exerc* 2016;48(7):1312-9. doi: 10.1249/MSS.0000000000000913

**Supplementary Table 1**

**Distribution of bouts of moderate to vigorous PA, light PA and SB, and breaks in sedentary time among British Men Without Pre-Existing CVD or Heart Failure, (n=1274).**

|  | Mean Minutes/day | % of total average daily wear time in bouts |
| --- | --- | --- |
| Bouts of MVPA (>1040 CPM) |  |  |
| 1-9 minutes | 30.3 | 3.5 |
| >=10 minutes | 10.0 | 1.1 |
| Bouts of LIPA (100-1040 CPM) |  |  |
| 1-9 minutes | 176.5 | 20.6 |
| >=10 minutes | 22.0 | 2.6 |
| Bouts of Sedentary behaviour (<100 CPM) |  |  |
| 1-15 minutes | 222.4 | 25.9 |
| 16-30 minutes | 128.3 | 15.0 |
| 31-60 minutes | 148.1 | 17.4 |
| >=61 minutes | 117.2 | 13.9 |

MVPA, moderate and vigorous physical activity, LIPA, light activity

**Supplementary Table 2**

**Akaike Information Criteria (AIC) for Linear Compared to Non-Linear Models of Physical Activity in Relation to All-Cause Mortality**

|  | Degrees of freedom | AIC | Degrees of freedom | AIC | Difference in AIC |
| --- | --- | --- | --- | --- | --- |
|  | Linear model | | Penalised spline model | |  |
| Steps/day | 1 | 2779 | 3 | 2747 | 32 |
| Sedentary minutes/day | 1 | 2779 | 3 | 2756 | 23 |
| Light PA minutes/day | 1 | 2782 | 3 | 2759 | 23 |
| MVPA minutes/day | 1 | 2793 | 3 | 2761 | 32 |

**Web Figure 1**

**Web Figure 2**
